# Tailoring Piezoelectric Nanogenerators and Microdevices for Cellular Excitation: Impact of Size and Morphology

**DOI:** 10.1002/advs.202415028

**Published:** 2025-02-14

**Authors:** Laura Lefaix, Marc Navarro, Carme Nogués, Andreu Blanquer, Gonzalo Murillo

**Affiliations:** ^1^ Institute of Microelectronics of Barcelona (IMB‐CNM, CSIC) Barcelona 08193 Spain; ^2^ Universitat Autònoma de Barcelona Bellaterra Barcelona 08193 Spain

**Keywords:** bioelectronics, cell stimulation, microdevice, nanogenerators, piezoelectrics

## Abstract

The use of piezoelectric devices as wireless electrical stimulators is an emerging research topic. In this study, piezoelectric microdevices, consisting of ZnO nanosheets (NSs) functioning as piezoelectric nanogenerators (NGs) grown on top of silicon microparticles, to electrically stimulate cell are designed. The morphology of the ZnO NSs is optimized by tuning the thickness of the aluminum nitride (AlN) catalyst layer and adjusting the growth duration. ZnO NSs grown on thinner AlN layers (≤ 200 nm) and subjected to 9 h of hydrothermal growth exhibit the most suitable characteristics for cell stimulation, balancing crystal size, and electric field generation. The generation of a local electric field capable of exciting osteoblast cells is inferred from finite element simulations and intracellular calcium influx measurements. The internalization rate of silicon microdevices of varying sizes (3 × 3, 6 × 10, 12 × 18 µm^2^) by osteosarcoma (Saos‐2) and primary human osteoblast (hOB) cells is assessed. The results show that smaller devices have higher internalization rates, particularly in tumoral Saos‐2 cells, while primary cells exhibit minimal internalization (< 10%) across all particle sizes. This study presents an optimized piezoelectric microdevice, based on a scalable and customizable fabrication process, for minimally invasive bioelectronic applications, offering accurate electrical cell stimulation while minimizing unwanted internalization.

## Graphical abstract

Piezoelectric microdevices, composed of ZnO nanosheets acting as nanogenerators and grown on silicon microparticles, are designed and fabricated to deliver precise electrical cell stimulation. This study optimizes nanosheet growth and microparticle size to enhance electrical cell activation performance while minimizing cellular internalization. These scalable microdevices show potential for promising applications in minimally invasive bioelectronics and wireless, targeted cell activation.

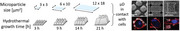

## 1 Introduction

Electroceuticals or bioelectronics, an emerging class of therapies that use electrical signals to treat diseases, are increasingly incorporating piezoelectric^[^ ^1^, ^2^ ^]^ for their ability to generate electric fields in response to mechanical stress, enabling precise, wireless stimulation of biological tissues.

The first piezoelectric nanogenerator (NG), developed in 2006,^[^ ^3^ ^]^ harnessed the piezoelectric effect to convert mechanical energy into electricity for powering nanodevices. Since then, piezoelectric NGs have been integrated into various technological areas, including energy harvesting, environmental monitoring, and medical applications. Among the potential applications of piezoelectric NGs, their integration in wireless battery‐less biomedical transducers has gained great interest in regenerative medicine. Wireless biomedical transducers are being developed for their use in neural,^[^ ^4^, ^5^, ^6^, ^7^ ^]^ muscle,^[^ ^2^ ^]^ bone,^[^ ^8^ ^]^ and skin^[^ ^9^ ^]^ stimulation and regeneration in the form of scaffolds, nanomaterials, and mm‐scale devices. Wearables implanted outside the body remain to be noninvasive and thus, the size of these devices is not a limiting factor. Nevertheless, to target inner tissues, the development of smaller implantable bioelectronics is essential.^[^ ^10^ ^]^

The work of Carmena et al. paved the way to develop wireless mm‐scale devices for stimulating electrically‐excitable cells.^[^ ^4^, ^5^, ^6^ ^]^ Their piezoelectric device, based on lead zirconate titanate (PZT), was reduced in size up to 1.7 mm^−3^ to create a system that would allow minimally invasive delivery methods. Shi et al., developed a similar device also composed of PZT and a volume of 0.065 mm^−3^ to stimulate the sciatic nerve.^[^ ^7^ ^]^ Through a wireless communication, they were able to deliver an electrical signal for nerve stimulation in a pre‐clinical stage. Nevertheless, the main limitations of their system were the presence of lead, which is toxic for the organism, and the millimetric spatial resolution.

Piezoelectric materials enable wireless energy transmission through ultrasounds, providing some advantages over other methods of wireless electrical stimulation. Due to the easy integration of certain piezoelectric materials into microelectromechanical systems (MEMS) technology, their size can be reduced up to cellular dimensions, providing the spatial resolution required for single‐cell stimulation.

ZnO nanosheets (NSs) have previously been demonstrated to function as piezoelectric NGs that induce cell stimulation.^[^ ^11^, ^12^ ^]^ Up to now, we have integrated ZnO NSs to obtain a minimally invasive lead‐free piezoelectric device at the micrometer scale, with a volume of up to 25 µm^3^, comparable to the size of a cell. It is based on the combination of silicon microparticles and ZnO NSs in a single microdevice. These ZnO NSs show a piezoelectric coefficient of 4–6 pm V^−1 [^ ^11^ ^]^ and a thickness of a few nanometers, making them highly responsive to bending stresses.^[^ ^13^ ^]^ Varying the dimensions of the ZnO NSs allows for the tuning of their electromechanical response and, thus, the electric field generated.^[^ ^14^, ^15^ ^]^

Additionally, our group has previously conducted a comprehensive biological^[^ ^11^, ^12^, ^16^ ^]^ characterization of the grown ZnO NSs using several types of cells. It is well known that cells vary in size and can exert different intensity of force on the piezoelectric materials. For instance, cells might respond differently to the same stimulus according to their lineage or type.^[^ ^17^ ^]^ For example, two different types of muscle cells, i.e., skeletal myotubes and smooth muscle cells, showed different responses to the same interaction with ZnO NGs.^[^ ^16^ ^]^ Compared to chondrocytes or osteoblasts, which are smaller and barely move, skeletal muscle cells are large and exhibit contraction movements.

To achieve effective cell stimulation, each piezoelectric microdevice must be customized. It has been reported that low‐magnitude electric fields (mV cm^−1^) and short times of stimulation (seconds) trigger the response in neurons.^[^ ^18^, ^19^, ^20^, ^21^ ^]^ Conversely, to induce the myogenic differentiation of mesenchymal stem cells (MSCs) high‐magnitude electric fields (up to hundreds of V/cm) for a short period of time (seconds) are required.^[^ ^22^ ^]^ For osteoblasts and epithelial cells, the electric fields must be higher than those for neurons, ranging from V/cm, with stimulation times varying from minutes to hours for osteoblasts ^[^ ^23^, ^24^, ^25^ ^]^ and a few minutes for epithelial cells.^[^ ^26^, ^27^ ^]^

Another important consideration is the size of the microdevice. Generally, small microparticles and submicroparticles, less than 1 µm^3^, are prone to be internalized by most of the cells, while larger microparticles can only be internalized by specialized cell types like macrophages and other specific cells, including tumoral cells.^[^ ^28^, ^29^, ^30^ ^]^ Therefore, if the goal is single‐cell stimulation, the microdevices should be larger enough, but still within the micrometer‐scale, to ideally reach the cell membrane without being internalized, thereby activating the signaling pathway from the outside of the cell.

**Figure 1:**
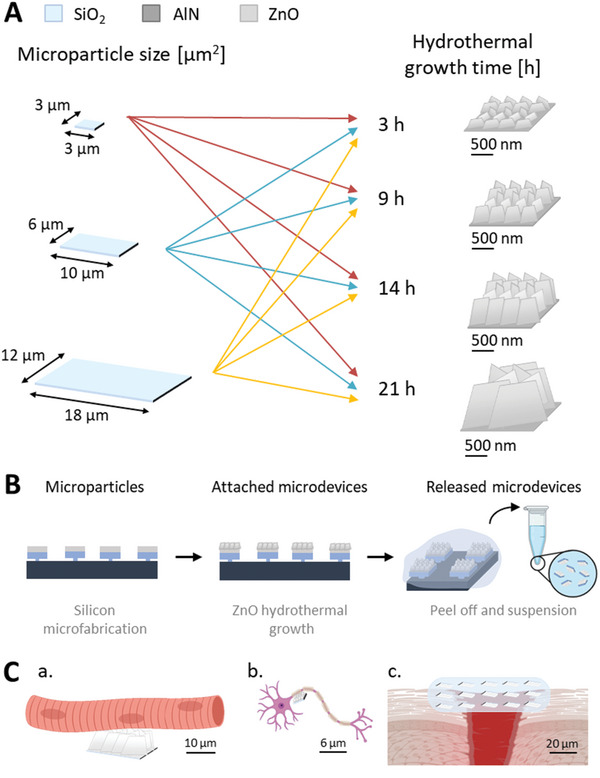
Design guidelines for the piezoelectric microdevices. A) Microparticle size (3 × 3, 6 × 10, 12 × 18 µm^2^) versus ZnO NS size depending on the duration of hydrothermal growth (3, 9, 14, 21 h). B) Fabrication of the microdevice. The steps that encompass the fabrication are: first, the manufacturing process of the microparticles in the cleanroom; second, the hydrothermal growth of the piezoelectric ZnO NSs, obtaining functional microdevices attached to the wafer; third, the peeling off and suspension process, ultimately leading to the released microdevices suspended in a biocompatible solution ready to be used in biological experiments. C) Potential biological targets and applications of microdevices, such as a) electrostimulation of muscle cells b) and neurons c) or wound healing.

The objective of this work is to develop microdevices specialized for each type of single‐cell stimulation. To achieve this, we have fabricated microdevices based on silicon microparticles with integrated ZnO NSs, varying two key parameters: the dimensions of the piezoelectric nanostructures and the size of the microparticle (**Figure** **1**). The former influences the microdevice's ability to electrically stimulate cells, while the latter affects the cells’ capacity to internalize or not the microparticle. To analyze the effect of the microdevices on the cell internalization, we tested two different cell lines: one derived from a cancer patient and the other from a healthy individual. The ultimate aim is to create a tunable device capable of adapting to the evolving requirements of future electroceutical applications.

## 2 Results and Discussion

Two distinct experimental blocks were conducted. The first focused on optimizing the ZnO nanostructured layer to enable electrical cell excitation, which was quantified through calcium detection. The second block aimed to determine the most suitable size of the microparticles, using cytocompatibility and internalization as the key parameters.

### 2.1 Optimization of the ZnO Nanosheets for Electrical Cell Excitation

The optimization of the ZnO nanostructured layer was conducted on glass coverslips sputtered with aluminum nitride (AlN) with different thickness. To grow the nanostructures, the hydrothermal method was used, which consists of an aqueous bath with Zn(NO_3_)_2_ and a basic buffer, such as hexametilentetramine (HMTA), at a controlled temperature.^[^ ^13^ ^]^

The presence of an aluminum‐based layer forms Al(OH)_4_^−^ ions in the solution, in this case due to the hydrolysis of AlN, which restricts the growth along the c‐axis, allowing the growth of thin ZnO NS crystals.^[^ ^13^ ^]^ Two main parameters could be tailored to optimize the morphology of the ZnO NSs: i) the thickness of the AlN layer and ii) the hydrothermal growth duration. The selection of these two parameters was based on previous studies which compared two different thicknesses of ZnO NSs and showed that the cell stimulation effect varied between thin (20.0 ± 1.3 nm) and thick (40.0 ± 4.5 nm) NSs, demonstrating that the NS geometry can be used to modulate the electric response elicited in the cell.^[^ ^11^ ^]^ The variability in the NS geometry was obtained by tuning the thickness of the AlN layer and the hydrothermal growth duration. Thus, this section presents an extensive study of how these two parameters influence the morphology of the ZnO NS layer.

**Figure 2:**
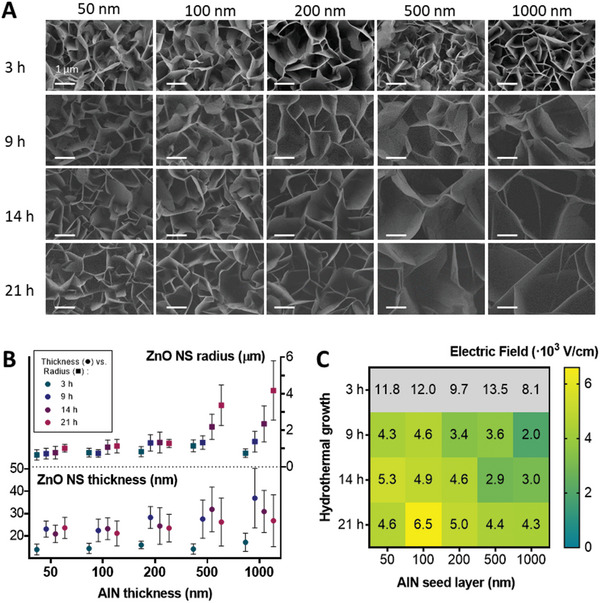
ZnO NS thickness and size variation depending on the hydrothermal growth parameters time and AlN layer thickness. A) SEM images of ZnO NSs. Rows correspond to different times (3, 9, 14, 21 h) of hydrothermal growth. Columns correspond to increasing thicknesses of the aluminum nitride (AlN) layer (50, 100, 500, 1000 nm). The scale bar in every image corresponds to 1 µm. B) Graph representing the ZnO growth radius and thickness quantified. C) Maximum electric field (V cm^−1^) produced by a single ZnO NS for each condition (growth duration and AlN layer thickness) when applying a force of 1 nN, as calculated by COMSOL Multiphysics simulations.

**Figure** **2**A shows the variation of the ZnO NS morphology (thickness and length) guided by the duration of the hydrothermal growth (rows) and the thickness of the AlN layer (columns) through scanning electron microscope (SEM) images. A solution of ZnNO_3_:HMTA (2:1) was used for each hydrothermal growth.^[^ ^13^, ^14^ ^]^ The formation of the nanostructures appeared to be thicker and larger with the increasing time of the hydrothermal growth and the increase of the AlN layer thickness. In the case of 1000 nm AlN and 21 h hydrothermal growth conditions, the result was a layer of well‐defined ZnO NSs of at least twice the dimensions obtained with shorter times and thinner AlN layers.

ZnO NS thicknesses and radius (i.e., side of the hexagonal crystal) were quantified and compared in Figure 2B. Both increased as time and AlN thickness increased. Looking specifically at the NS radius, for thinner layers (50, 100 nm AlN), they were smaller than 1 µm and barely changed. For 200 nm AlN, the highest mean radius obtained was 1.3 µm after 9 h or more hours of hydrothermal growth. For 500 and 1000 nm thick AlN, a 9 h growth limited the radius to 1.3 µm, but longer times of hydrothermal growth allowed to attain a radius size of 2 µm after 14 h and of 3–4 µm after 21 h.

Regarding the thickness of the NSs, it was quite homogeneous for thinner AlN layers, while more heterogeneous for the thicker ones tested. This tendency is depicted in the NS thickness histograms of Figure S1 (Supporting Information). The values for the mean thickness of the ZnO NSs, quantified for every condition studied, ranged from 14 and 37 nm. The highest mean thickness, 37 ± 13 nm, was observed after 9 h of hydrothermal growth with 1000 nm of AlN layer thickness. The smallest mean thickness, 14 ± 2 nm, was obtained after 3 h of hydrothermal growth with AlN layer thicknesses of 50, 100 and 500 nm. This suggests a direct correlation between the thickness of the ZnO NSs and the growth duration, as long as Al(OH)_4_^−^ ions are available in the solution.

Very short growth times (3 h or less) result in extremely thin ZnO NSs with only a few nanometers of crystal thickness, which means insufficient development (with a potential diminishing of the piezoelectric coefficient) and NS collapse after sample drying. This NS collapse was observed during standard SEM inspection, resulting in folded NSs with an apparent thickness doubling the actual value. It required the use of hexamethyldisilane (HMDS) as an efficient drying method to minimize surface tension during the liquid‐to‐gas transition. This method enables the acquisition of accurate SEM images for measuring NS size and analyzing morphology (Figure S2, Supporting Information). However, because the sample is subjected to different rinsing and drying steps, NS collapse cannot be discarded, making these samples, in principle, not intended for reproducible stimulation applications in biological experiments.

Simulations of the electric field that a single ZnO NS can generate were performed for each condition tested. The aim was to obtain the maximum theoretical value of electric field intensity that different sizes of ZnO NS are able to produce by applying a force of 1 nN in the upper border of the nanostructure. The resulting electric field intensities produced are depicted in Figure 2C.

As shown in the heatmap, for all the AlN layer thicknesses, as the growth time increases, the ZnO NSs become better defined, leading to an increase in the electric field produced. For larger nanostructures formed on thicker AlN layers (500 and 1000 nm), the electric field generated is lower than for nanostructures formed on thinner AlN layers (50 and 100 nm). This reduction in the electric field is attributed to a decrease in the moment of inertia when a force is applied at the edges of the larger NSs. Therefore, there is a tradeoff between the NS thickness and length, being the best option the one with well‐defined crystals but a moderated aspect ratio. As expected, theoretical high electric field values were obtained for 3‐h hydrothermal growth for all the AlN layer thicknesses. However, as previously mentioned, we hypothesize that the 3‐h growth resulted in limited crystal size, insufficient development, and uncontrollable NS collapse after sample drying, which can have a significant impact on the electric field generated. Despite these issues, these extremely thin structures were tested for later cell stimulation experiments, considering that the theoretical values of electric field intensity could be reduced.

Considering the measured values of NS thickness and radius, and the theoretical maximum intensity of the generated electric field, an interesting strategy is to reduce as much as possible the AlN thickness and the growth time to increase the simplicity and cost of the synthesis but maintaining well‐defined crystals and large enough electric field magnitudes. All these conditions are achieved after at least 9 h of hydrothermal growth with thin AlN layers (less than 200 nm).

**Figure 3:**
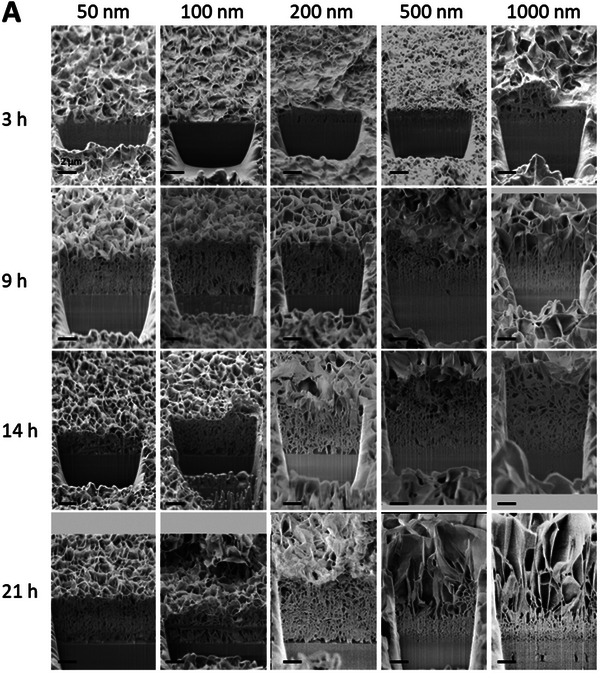
FIB micro‐sectioning of the ZnO NSs layer was obtained from the different AlN thickness and hydrothermal growth time.

Using the focus ion beam (FIB) technique, micro‐sectioning from several samples of each condition were studied to understand the variation in the ZnO NS morphology. The growth of ZnO NSs occurred through the superposition of several crystal layers, one on top of the others, increasing in the NS length and thickness at each new layer (**Figure** **3**).

We analyzed the cell response to these electric fields generated by the different sizes of ZnO NSs, when growing osteoblast on top of them. We hypothesized in a previous study that the movement and the attachment of osteoblasts on top of ZnO NS were able to bend the NS and as a consequence, due to the piezoelectric effect, to produce an electric field around the plasma membrane that in turn trigger the opening of the calcium channels.^[^ ^11^ ^]^ These electromechanical interactions among ZnO NSs and osteoblasts were studied through the intracellular calcium changes over time.

Among all the conditions for AlN thickness and hydrothermal growth time, four different conditions were selected to be tested in cell cultures. Three were obtained with an AlN thickness of 100 nm (3, 9, and 21 h of hydrothermal growth) because of the proper increasing electric field intensities calculated, and one obtained with a thickness of 500 nm (21 h of hydrothermal growth) presenting the higher electric field intensity obtained among the thicker AlN layers. The conditions tested for growth time and AlN layer thickness were: i) control glass coverslip without piezoelectric ZnO NSs, ii) 3 h and 100 nm AlN, iii) 9 h and 100 nm AlN, iv) 21 h and 100 nm AlN, and v) 21 h and 500 nm AlN. The thickness and radius mean values of the ZnO NSs measured for each condition were: ii) 14 ± 2 nm and 800 nm ± 200 nm, iii) 22 ± 5 nm and 700 ± 200 nm, iv) 21 ± 6 nm and 1100 ± 400 nm and v) 26 ± 11 nm and 4000 ± 1000 nm, respectively.

**Figure 4:**
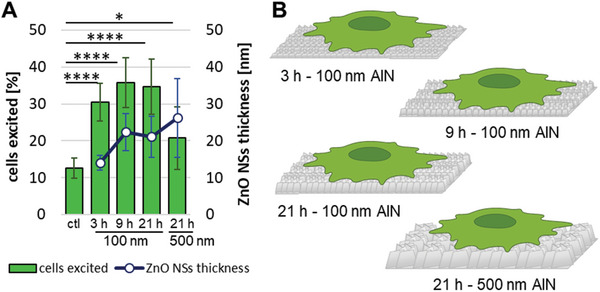
Effect of the morphology of ZnO NS on cell excitation. Increase of intracellular calcium content as a response of the electrical excitation produced when cells exert force on the piezoelectric ZnO NSs. A) Percentage of cells excited per each condition tested: for 100 nm of AlN, 3 h, 9 h and 21 h; for 500 nm AlN, 21 h versus control (ctl) without piezoelectric nanostructures. B) Scheme representing a Saos‐2 cell (15 to 20 µm) scaled with respect to the nanostructures.

In **Figure** **4**A, the percentages of excited cells are depicted. For the different ZnO NSs studied, the percentage of excited cells was 31% ± 5% (3 h, 100 nm), 36% ± 7% (9 h, 100 nm), 35% ± 8% (21 h, 100 nm) and 21% ± 9% (21 h, 500 nm) with respect to the control (13% ± 3%). No significant differences were found in the percentage of cells excited among the samples with 100 nm of AlN with different of hydrothermal growth. Even for the 3‐h hydrothermal growth substrates, the response was similar to the rest of the substrates grown on 100 nm of AlN, dismissing the values simulated in Figure 2C and supporting our hypothesis of poorly‐developed crystals and the potential collapse of the NSs, effectively doubling their actual thicknesses and creating a charge compensation between the two opposite‐facing surfaces of the folded NSs (i.e., ultimately decreasing the expected electric field magnitude). By contrast, significant differences were found between each 100 and 500 nm samples analyzed and the control sample. These differences support the assumption that cell excitation is induced due to the piezoelectric nature of the ZnO NSs, which produces a local electric potential in response to the force exerted by the cell when moving or attaching to the material. The dimensions of the different ZnO NSs with an AlN layer of 100 nm were very close and the percentage of cells excited remained between 31% and 36% for all cases. However, the ZnO NSs grown after 21 h with an AlN layer of 500 nm were thicker than those from the 100 nm samples, and the percentage of excited cells was sensibly lower. We hypothesize that these thicker nanostructures are more difficult to bend for a certain cell force, due to a larger effective elastic constant, (Figure 4B) resulting in a decreased percentage of excited cells in the sample. Significant differences in the percentage of cells excited were also obtained among the 500 nm samples and the control, supporting again the assumption that the piezoelectric ZnO NSs are responsible for the percentage of cells excited. In conclusion, for these conditions, the percentage of cells excited increases as the size of the ZnO NS decreases.

As demonstrated in this experimental block, ZnO NSs obtained after 9 h of hydrothermal growth on an AlN layer with a thickness of 100 nm depict a good compromise in between NS length and thickness. In addition, this condition gets the highest percentage of excited cells. Longer times of hydrothermal growth would promote a similar response, although a reduction in the growth time would simplify the fabrication process.

### 2.2 Influence of Microdevice Size on Cellular Internalization

After testing the electrical performance of piezoelectric ZnO NSs under various growth conditions, this study aimed to integrate these nanostructures onto silicon‐based microparticles of different sizes to produce the final microdevices. We then analyzed how the size of these microdevices affects their internalization by cells.

**Figure 5:**
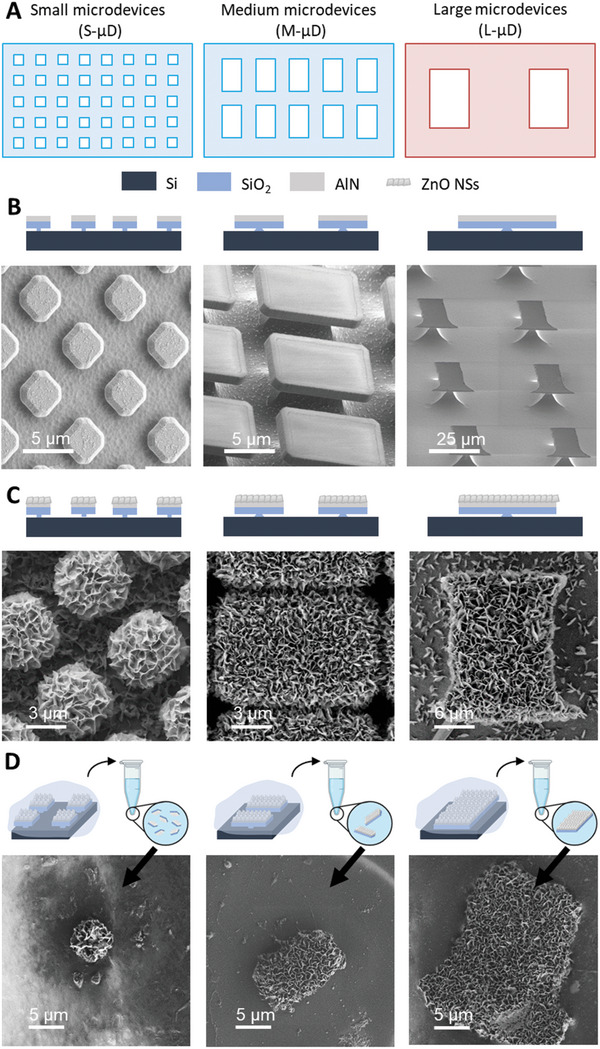
Fabrication of microdevices of different sizes. The images are organized per columns arranged by the size of the microdevices (µD) fabricated, small, S‐µD; medium, M‐µD; and large, L‐µD. A) Comparative scheme of the mask used to define the area of the microparticles, 3 × 3, 6 × 10^,^ and 12 × 18 µm^2^, respectively. B) Schematic cross‐section of the microparticles obtained after completing the microfabrication process, along with SEM images of the fabricated microparticles tilted at 45°. C) Schematic cross‐section of the microdevices after the hydrothermal growth, along with SEM images of the fabricated microdevices, attached to the substrate wafer, featuring the piezoelectric ZnO NS layer. D) Scheme of the peeling‐off process, showing the detachment of the microdevices from the wafer and the dissolution of the polymer embedding them, accompanied by SEM images of the resulting released microdevices.

The design of the microdevices’ size is limited by the later application, as a single‐cell spatial resolution is aimed. This resolution is achievable for microdevices of a few microns in size. On the other hand, reducing the size of the microdevices in excess can lead to other potential issues, as an excessive uptake could negatively impact on the cell survival.^[^ ^28^ ^]^ Our preliminary results demonstrated that microparticles up to 3 µm in size, made of silicon and polysilicon, were cytocompatible.^[^ ^29^, ^30^ ^]^ Therefore, microparticles with three different sizes, in a reasonable range, were fabricated, i.e., small, S (3 × 3 µm^2^), medium, M (6 × 10 µm^2^), and large, L (12 × 18 µm^2^) sizes (**Figure** **5**A).

Two different photolithographic masks along with a mask‐less laser writing design were used to produce three sizes of microparticles (Figure 5B–C). The size obtained after the microfabrication process was of 3.58 ± 0.05 µm x 3.56 ± 0.05 µm (S), 6.68 ± 0.06 µm × 10.96 ± 0.06 µm (M), and 13.5 ± 0.6 µm × 19.3 ± 0.3 µm (L), respectively (Figure 5D).

The microparticles created using the mask‐less laser writing were less homogeneous than the ones obtained by mask photolithography and showed the largest difference with respect to the original design – one micron. The advantage of this mask‐less technique is the process flexibility and fast customization of the design.

The micropatterns fabricated using the mask aligner had a small standard deviation in the measured length, indicating a great homogeneity on the wafer. Thus, the microfabrication based on mask aligners becomes cost‐effective and highly reproducible.

The nanostructured piezoelectric layer of ZnO NSs was grown on top of the microparticles using the aluminum‐based AlN surface layer as a crystal layer together with the ZnNO_3_:HMTA solution.^[^ ^13^, ^14^ ^]^ We used hydrothermal growth for 9 h an AlN layer of 100 nm of AlN based on the optimization of the parameters of previous experiments (Results Section 2.1) and studies in our group. However, the ZnO NS thickness and radius also varied from S‐ to M‐ and L‐microdevices (µD) for these established parameters. After 9 h of growth, the mean NS thickness measured for S‐µDs was of 32 ± 4 nm, for M‐µDs of 23 ± 5 nm, and for L‐µDs of 17 ± 3 nm. These differences in the ZnO NS size might be influenced by the AlN layer area exposed to the solution during the hydrothermal growth, as the time and thickness of the layer are the same in every case.

The final sizes measured for the microdevices were 4.9 ± 0.3 µm x 5.0 ± 0.3 µm for S‐µDs, 8.9 ± 0.2 µm x 12.8 ± 0.2 µm for M‐µDs and 18.2 ± 0.8 µm x 25.5 ± 0.8 µm for L‐µDs (Figure 5E). This growth ended up with a top layer of ZnO NSs over the S‐, M‐, and L‐microparticles with a thickness of 1.5, 2, and 6 µm respectively.

The microdevices (i.e., the combination of the silicon microparticle with the ZnO NSs coverage) were peeled off to release them from the wafer (Figure 5F; Figure S3, Supporting Information). After the peel‐off step, an 82% of the S‐µDs were recovered. The recovery was below the 30% for the M‐µDs (29%) and L‐µDs (26%). In the case of M‐µDs, the short spacing (1:2) between the microparticles defined in the pattern was a determinant for the recovery. Many of them were attached to each other after the hydrothermal growth as the spacing was insufficient to obtain local ZnO NSs growth and, therefore, discrete microdevices. This attachment made it more difficult for the Fluoromount polymer to infiltrate the gaps between them, thereby hindering the release of these microdevices. This means that the distance between adjacent microdevices (i.e., pitch) has to be designed large enough to obtain single microdevices (as in the case of S‐ and L‐µDs) instead of aggregates (M‐µDs) while keeping this pitch as reduced as possible to maximize the number of microdevices per wafer (i.e., increasing the overall yield). For L‐µD, the low recovery efficiency was attributed to the overlapping of the pattern in certain areas of the chip during the direct laser writing step, which left an overexposed AlN pathway that interconnected some microdevices.

Once determined how the size of the microparticles is modified by the hydrothermal process, we tested the cytocompatibility and the capacity of the cells to internalize S‐, M‐, and L‐µDs. It is already known that microparticles can be internalized by cells. However, the percentage of internalization could change according to the size of microparticles and the cell type.^[^ ^29^, ^30^, ^31^ ^]^

Our goal is the activation of the voltage‐gated calcium channels (VGCCs) present at the cell membrane by creating a local electric field with piezoelectric microdevices. The unnecessary internalization of these microdevices could cause dysregulation of intracellular metabolic pathways. Thus, to prevent internalization, we studied two parameters that can influence internalization. The first one, is microdevice size. Larger microdevices (M‐ and L‐µDs), harder for the cell to internalize, were fabricated. Additionally, we tested if the use of a primary cell culture would prevent internalization due to the correct regulation of the uptake mechanisms that might be altered in the Saos‐2 cell line. Two different types of bone cells were used for this purpose: an osteosarcoma cell line (Saos‐2; isolated from a cancer patient), as in previous experiments, and an osteoblasts primary cell culture (hOB; isolated from a healthy patient).

**Figure 6:**
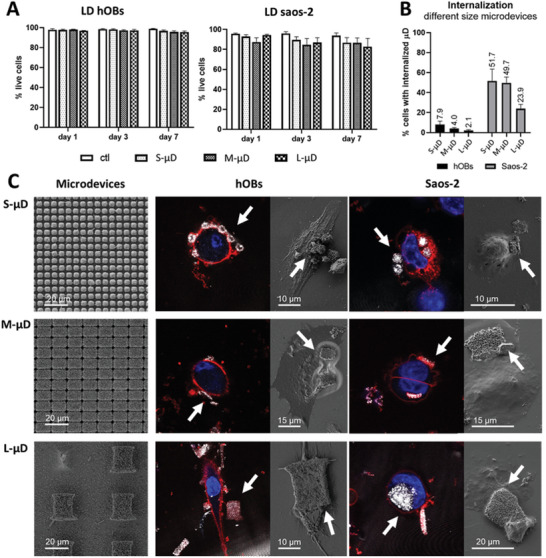
Cytocompatibility and internalization study of microdevices of different sizes. A) Cytocompatibility of two types of cells, hOB (primary cell culture) and Saos‐2 (cancer cell line) incubated in the presence of microdevices (S, M, L‐µDs) for 1, 3, and 7 days, B) Percentage of internalization of different size microdevices for the two types of cells. C) Images of the different size microdevices (S, M, L‐µD) with respect to hOB cells and Saos‐2 cells. Organized by column: first, SEM images of the microdevices before detaching them from the wafer; second, confocal laser scanning microscope (CLSM) and SEM images of the different size microdevices in contact with hOB cells; third, CLSM and SEM images of the microdevices in contact with Saos‐2 cells. In CLSM images, cell membranes are stained with cell mask (red) and nuclei with Hoestch (blue); white arrows point at the released microdevices.

Viability results for primary cells hOB at days 1, 3, and 7 cultured in the presence of any microdevice was above 95% for all time‐points analyzed. In Saos‐2 cells, the viability percentage for L‐µDs was reduced to 80%. Thus, microdevices could be considered cytocompatible according to ISO 10993–5 (**Figure** **6**A), although Saos‐2 cells showed a reduction in viability compared to hOB.

The results obtained for the cytocompatibility of the microdevices appear to be directly related to the internalization results, depicted in Figure 6B. Internalization is analyzed after 24 h of being in contact with the microdevices with the cell culture. Reproducing the result obtained in previous experiments, around 50% of S‐µDs were internalized (52%) in Saos‐2 cell cultures. Regarding the larger microdevices, M‐µDs were internalized up to 50% and L‐µDs the half, 24%. On the contrary, in hOB cells, for any microdevice size analyzed, the percentage of internalization was below 10%. For S‐, M‐ and L‐µDs only 8%, 4%, and 2% of the microdevices were internalized, respectively. As expected, in both cellular types, the internalization was inversely proportional to the size of the microdevice.

The results proved that, in non‐tumoral cells, the internalization was far less than the obtained for tumoral cells, Saos‐2 cells. It has been reported that the internalization of microdevices larger than 200 nm requires a specific endocytic mechanism named macropinocytosis. In tumoral cells, macropinocytosis is usually enhanced, which allows the uptake of macromolecules, nutrients, or other types of particles.^[^ ^32^, ^33^ ^]^ This likely explains the increased internalization observed in the Saos‐2 cell line. Moreover, by increasing the size of the microdevices, internalization decreased. Comparing S‐ and L‐µDs, in tumoral cells the internalization percentage was reduced to half and, in non‐tumoral cells, up to a 75%. Cytocompatibility and internalization appear to be related, as, when the internalization decreased (hOB, less than a 10%), cytocompatibility increased.

In Figure 6C, CLSM and SEM images allowed us to compare the microdevice size with respect to the cell. hOB cells are approximately twice the size of Saos‐2 cells. Despite this, in the hOB cell images, the microdevices are located outside the cells, in contact with the cell membrane, as seen in both images. Conversely, in the CLSM images of Saos‐2 cells, half of the microdevices in each image, regardless of size, are internalized, while the other half remain outside. These are representative images of the results quantified for Figure 6B.

As previously mentioned, the positioning of the microdevices is critical for their effectiveness. The electric field generated by a microdevice, upon actuation, should be localized near the plasma membrane, ideally outside the cell, to rearrange the movable ions in the extracellular medium. This charge rearrangement creates a local potential difference across the membrane, capable of activating voltage‐dependent ion channels. The piezoelectric microdevices were introduced into the cell culture medium, where they settled on the adhered cells by precipitation. After 24 h, results indicated that most microdevices were in close contact with the cells. Notably, non‐tumoral cells interacted with the plasma membrane without internalizing the microdevices. Therefore, these fabricated microdevices can be considered potential tools for electrically stimulating non‐tumoral cells, regardless of the microdevice size.

The potential biomedical uses of these microdevices are extensive and include various wireless minimally invasive applications. For instance, microdevices could interact with target cells to induce an electric field that self‐stimulates cellular activities such as cell migration or muscle contraction, or they could be driven by wireless actuation, such as ultrasounds or magnetic fields. In both scenarios, the electric field generated by the microdevices would be localized at the cellular level, enabling minimally invasive cell stimulation.

## 3 Conclusion

In this work two different parameters were tailored to create piezoelectric microdevices for cell stimulation: the length and thickness of the ZnO NS and the size of the microparticle supporting these NSs. By modifying both parameters, we have created a microdevice capable of electrically exciting cells from the outside of the cell membrane. First, a dependence on the time of the hydrothermal growth and the thickness of the AlN layer affecting the dimensions of the ZnO NSs was demonstrated. Besides, how the dimensions of the ZnO NSs can modify their performance as nanogenerators related to the cell excitation through calcium fluxes was exhibited as well. Among the samples tested, the small ZnO NSs induce larger responses in the osteoblast population. We hypothesize that these small ZnO NSs are easier for osteoblast cells to bend, causing a larger strain, for a determined force exerted. The integration of the ZnO NSs on microparticles of different sizes was studied, also resulting in the fabrication of microdevices of different sizes. The modification of some steps during the microfabrication process allowed us to optimize some parameters such as the spacing needed between microparticles to ensure proper peeling off and recovery. Finally, microdevices of different sizes were tested for internalization in bone tumoral cells and bone primary cells, demonstrating that as the size increases, the internalization is reduced. However, the most interesting result obtained after the internalization study was that primary bone cell cultures barely internalized microdevices (less than 10%) with respect to the tumoral cells (50% for S‐µDs).

## 4 Experimental Section

### Hydrothermal Growth of ZnO Nanosheets

Glass coverslips with a diameter of 12 mm, attached to a silicon wafer, were sputtered (Leybold Heraeus Z‐550‐SM) to deposit varying thicknesses of AlN (50, 100, 200, 500, 1000 nm). For each thickness of the AlN layer, different times of hydrothermal growth were set (3, 9, 14, and 21 h). Four coverslips were placed in a sealed container with a ZnNO_3_·6(H_2_O):HMTA (2:1) solution in an oven at 80 °C. Samples were rinsed with distilled water for 1 min followed by a rinsing with ethanol 100%, also for 1 min. The rinsing was used to prevent the collapse of the nanostructures due to the lower surface tension of ethanol. For samples with 3 h of hydrothermal growth, after checking the rinsing with ethanol was insufficient to prevent the collapse of these nanostructures when drying, samples were dried out in HMDS for 45 min to ensure faithful SEM images. SEM (Auriga‐40, Carl Zeiss) was used to characterize the ZnO NSs layer grown using a magnification ranging from 100 to 50kX and the secondary electron (SE2) detector at 2 keV. Images of 50kX were used for measuring diameters and thicknesses of ZnO NSs using ImageJ software (*n* = 15). The NS radius was equivalent to the side length of the ZnO crystal hexagon, which was also the radius of the circumscribed circle.

### COMSOL FEM Simulations

COMSOL Multiphysics 6.0 (COMSOL, Sweden), a 3D finite element software, was used to simulate the electric field by a single ZnO NS under a 1 nN force exerted on the upper edge of the nanostructure. The dimensions of the ZnO NS were defined in every case based on the measurements performed by SEM. In the model, a ZnO NS was anchored to the microparticle defining the bottom as a fixed constraint. The upper edge of the ZnO NS was free to move. An anisotropic tetrahedral mesh was created to improve the convergence and solver efficiency. For the smallest ZnO NS (3 h of growth, 50 nm of AlN thickness) the minimum element size in the mesh was 26.3 nm, ZnO NS, and the maximum one, microparticle, was 210 nm. For the largest one (21 h of growth, 1000 nm of AlN thickness), 50.2 and 690 nm were the minimum and maximum element sizes respectively.

### Cell Culture

Human osteosarcoma cells, Saos‐2, purchased from the American Type Culture Collection (ATCC HTB 85), were cultured in Dulbecco Modified Eagle Medium (DMEM) supplemented with 10% fetal bovine serum (FBS) and standard conditions (5% CO_2_, 37 °C). Human osteoblasts, hOBs, were obtained from healthy human donors. These cells were cultured in DMEM supplemented with 20% FBS and 2% streptomycin in standard conditions.

### Cell Excitation Experiments

The induction of calcium transients in Saos‐2 cells cultured on top of glass coverslips with ZnO NS layers (100 nm of AlN layer thickness, 3 h, 9 h, 21 h, and 500 nm of AlN layer thickness, 21 h of hydrothermal growth conditions) was studied. For the intracellular calcium measures, 5 ×10^4^ Saos‐2 cells per well were seeded on top of a glass coverslip of 12 mm diameter without (control) or with ZnO NSs in a four‐well plate. They were cultured for 24 h to allow cell adhesion. After 24 h, samples were prepared for their examination under the CLSM (SP5, Leica). Samples were washed and incubated with serum‐free medium containing 1 µLmL^−1^ FLUO‐4 calcium dye (Thermo Fisher Scientific) for 30 min and 37 °C in darkness. Once the incubation was completed, the medium was removed, and the cells were washed again. Finally, coverslips with cells were placed in a glass‐bottom petri dish and incubated in phenol red free DMEM supplemented with 10% FBS for 10 min at 37 °C. At the CLSM, tempered at 37 °C, the sample was placed upside‐down with the cells facing the glass‐bottom of the petri dish. Time lapse images were taken for 15 min. A 10× objective was used. The number of cells studied per condition was at least 200 cells. The time lapse images were analyzed, and the number of intracellular calcium peaks was quantified.

### Fabrication of the Microparticles

The overall process was developed in the microfabrication clean room at the IMB‐CNM (CSIC). First, the run was designed with a program where every step was detailed, based on previous works.^[^ ^34^ ^]^ Then, 4‐inch silicon wafers were used as substrates and, after the corresponding cleaning, a layer of 1 ± 0.4 µm of silicon dioxide (SiO_2_) was grown in the oven (TS Series V, Tempress) at a temperature of 1100 °C. The following step was the metallization of the samples with AlN, sputtered (Leybold Heraeus Z‐550‐SM) with a thickness of 100 nm. The photolithography step included the use of a positive resist (HiPR 6512 photoresist) with a thickness of 1.2 µm for all the metalized wafers with 100 nm of AlN. Three different patterns were used: one defining an area of 3 × 3 µm^2^ spaced by 3 µm of distance (1:1), a second pattern creating 10 × 6 µm^2^ microparticles with 3 µm of separation (2:1), both using a Karl Süss MA6 mask aligner; a third pattern of 18 × 12 µm^2^ separated from the others by a distance of 36 µm in the larger border and 24 µm in the shorter one (1:2). This mask‐less pattern was defined by using the laser writing equipment (KLOÉ Dilase 650). of Afterwards, an isotropic etching of the full 100 nm AlN layer was performed with a chlorinated mixture for 75s (SI 500 ICP‐RIE plasma etcher, SENTECH). The photoresist was annealed for 30 min at 200 °C. Using the resist deposited and the metal beneath it as a mask, the isotropic dry etching of SiO_2_ (AMI ETCH P‐5000) was performed in the wafers with 100 nm of AlN for 200 min. The resist was stripped after the etching. After this, a silicon pillar under the microparticles was created by anisotropic dry underetching (DRIE ALCATEL 601E) for 25 s (3 × 3 µm^2^ area micropattern), 40 s (10 × 6 µm^2^ area micropattern) and 55 s (18 × 12 µm^2^ area micropattern). The pillar left was calculated to be 1/3 of the microparticle size. Each wafer was examined using SEM (Auriga‐40, Carl Zeiss) and confocal microscopy to determine the final size of the microparticles and their supporting pillar. After the entire process, one wafer was obtained for each pattern of 3 × 3 µm^2^, small (S), 10 × 6 µm^2^, medium (M), and 18 × 12 µm^2^, large (L). All the resulting three wafers had an AlN layer of 100 nm and an underneath SiO_2_ layer of 1 µm.

### Hydrothermal Growth of ZnO NSs on Top of the Microparticles

The wafers were diced into 1 × 1 cm^2^ size chips. The chips were placed upside down in a sealed container suitable for their size. Then, they were covered with a ZnNO_3_·6(H_2_O): HMTA (2:1) solution and placed in an oven at 80 °C for 9 h. These hydrothermal growth conditions allowed ZnO NSs to grow on the chip surface thanks to the catalyst effect of the AlN layer. After the chemical reaction took place, the chips were removed from the container. They were rinsed first in deionized water, and then in ethanol for a minute each. Later, the chips were left to dry at room temperature and stored. SEM allowed the characterization of the morphology of ZnO NSs. Parameters such as thickness, radius, and surface covering were measured for a magnification range from 100 to 50kX using the SE2 detector at 2 keV.

### Microdevice Peeling‐Off

The chips produced after the hydrothermal growth were attached to a glass slide with double‐faced scotch tape and covered completely with Fluoromont aqueous mounting medium. After drying overnight, the polymeric layer was pulled out, detaching the microdevices from the substrate, as first described in ref. [34]. Later, 1 mL of deionized water was added to dissolve the polymer and left for 3 h to allow the precipitation of the microdevices. Several washing steps were performed to remove the Fluoromont polymer, leaving time after each washing for the microdevices to precipitate. Finally, the microdevices were resuspended in ethanol to preserve them from contamination. Before each experiment, the ethanol was removed and the microdevices were resuspended in the cell medium. The concentration of each batch was quantified in a Neubauer chamber.

### Cytocompatibility and Internalization Cell Culture Experiments

For the viability experiments, either 5×10^4^ Saos‐2 cells per well or 2 ×10^3^ hOB cells were seeded in a 35‐mm petri dish with a 2‐well culture insert (IBIDI, Germany) and they were cultured for 24 h to allow cell adhesion. After the adhesion, 1·× 10^5^ S‐ or M‐µDs or 5·× 10^4^ L‐µDs were added to one of the wells in Saos‐2 cell cultures. For hOB cells, either 4·× 10^3^ S‐ or M‐µDs were added or 2·× 10^3^ L‐µDs. The proportion for S‐µDs and M‐µDs between microdevices and cells was 2:1. For L‐µDs, the proportion between microdevices and cells was 1:1. Samples were then evaluated after 1, 3, and 7 days. To assess the cytotoxic effect of microdevices, live/dead staining containing calcein (0.5 µLmL^−1^) and propidium iodide (2 µLmL^−1^) was used. The culture plate was incubated at 37 °C for 15 min in darkness and, later, observed under an inverted fluorescence microscope (Olympus). Eight images from different fields were taken. Then, ImageJ cell counter (NIH) was used to process the images. CLSM was used to quantify the microdevice internalization for the two types of cells. Cells with microdevices were cultured for 24 h in the same conditions previously described for viability experiments. Then, samples were stained with 1 µg mL^−1^ of cell mask plasma membrane live stain (Invitrogen) and 1 µg mL^−1^ of Hoechst 33258 (Invitrogen). A 63X oil immersion objective was used to collect horizontal optical sections. Microdevices were studied through reflection lenses. For each individual cell, the z‐stack was evaluated. The microdevice position was assessed by correlating the higher plane of the membrane, corresponding to the limit of the cell membrane, and the higher plane of the microdevice analyzed, corresponding to the limit of the microdevice. At least, 100 cells per sample were analyzed. SEM was used to assess the position of the microdevices with respect to the cells. Samples were fixed with 4% paraformaldehyde (PFA) for 15 min and dehydrated using increasing concentrations of ethanol (30, 50, 70, 90, 100%) for 15 min. After that, samples were dried using HMDS for 45 min. The magnification of the images ranged from 100 to 50kX at 2 keV.

### Statistics

Statistical software (GraphPad, Prism 8.0.2) was used to conduct all the statistical analyses. The difference between groups was obtained by performing t‐student tests for the results of the calcium peak experiment, considering *p* < 0.05 (^*^) as the minimum statistical difference level. One‐way ANOVA test was performed to analyze the statistical difference between the groups correlating cell cytocompatibility and the size of microdevices. The statistical significance was considered above p < 0.05 (^*^) in both. All the biological experiments statistically analyzed included at least three replicates.

## Conflict of Interest

The authors declare no conflict of interest.

## Supporting information



Supporting Information

## Data Availability

The data that support the findings of this study are available on request from the corresponding author. The data are not publicly available due to privacy or ethical restrictions.

## References

[1] H. Xue , J. Jin , Z. Tan , K. Chen , G. Lu , Y. Zeng , X. Hu , X. Peng , L. Jiang , J. Wu , Sci. Adv. 2024, 10, eadn0260. 38820150 10.1126/sciadv.adn0260 PMC11141629

[2] R. Qiu , X. Zhang , C. Song , K. Xu , H. Nong , Y. Li , X. Xing , K. Mequanint , Q. Liu , Q. Yuan , X. Sun , M. Xing , L. Wang , Nat. Commun. 2024, 15, 4133. 38755124 10.1038/s41467-024-48468-x PMC11099052

[3] Z. L. Wang , J. Song , Science 2006, 312, 242. 16614215 10.1126/science.1124005

[4] D. Seo , R. M. Neely , K. Shen , J. M. Rabaey , J. M. Carmena , M. M. Maharbiz , D. Seo , R. M. Neely , K. Shen , U. Singhal , E. Alon , J. M. Rabaey , J. M. Carmena , Neuron 2016, 91, 529. 27497221 10.1016/j.neuron.2016.06.034

[5] R. M. Neely , D. K. Piech , S. R. Santacruz , M. M. Maharbiz , J. M. Carmena , Curr. Opin. Neurob. ‐ScienceDirect 2018, 50, 64. 10.1016/j.conb.2017.12.010 29331738

[6] D. K. Piech , B. C. Johnson , K. Shen , M. M. Ghanbari , K. Y. Li , R. M. Neely , J. E. Kay , J. M. Carmena , M. M. Maharbiz , R. Muller , Nat. Biomed. Eng. 2020, 4, 207. 32076132 10.1038/s41551-020-0518-9

[7] C. Shi , V. Andino‐Pavlovsky , S. A. Lee , T. Costa , J. Elloian , E. E. Konofagou , K. L. Shepard , Sci. Adv. 2021, 7, eabf6312. 33962948 10.1126/sciadv.abf6312 PMC8104878

[8] C. Xi , X. Lingling , S. Yizhu , J. Li , J. Jianying , E. Wang , B. Zhang , X. Wen , Y. Bai , D. Luo , C. Chen , Z. Li , Sci. Bull. 2024, 69, 12. 10.1016/j.scib.2024.04.002 38637224

[9] J. Shi , S. Kim , P. Li , F. Dong , C. Yang , B. Nam , C. Han , E. Eig , L. L. Shi , S. Niu , J. Yue , B. Tian , Science 2024, 384, 1023. 38815037 10.1126/science.adl1102

[10] A. Khalifa , S. Lee , A. C. Molnar , S. Cash , Bioelectron Med. 2021, 7, 19. 34937565 10.1186/s42234-021-00080-w PMC8697496

[11] G. Murillo , A. Blanquer , C. Vargas‐estevez , L. Barrios , E. Ibáñez , C. Nogués , J. Esteve , Adv. Mater. 2017, 29, 1605048 10.1002/adma.201605048 28437016

[12] O. Careta , J. Fornell , E. Pellicer , E. Ibañez , A. Blanquer , J. Esteve , J. Sort , G. Murillo , C. Nogués , Biomedicines 2021, 9, 352. 33808338 10.3390/biomedicines9040352 PMC8065972

[13] G. Murillo , I. Rodríguez‐Ruiz , J. Esteve , Cryst. Growth Des. 2016, 16, 5059.

[14] G. Murillo , E. Leon‐salguero , P. R. Martínez‐alanis , J. Esteve , J. Alvarado‐rivera , F. Güell , Nano Energy 2019, 60, 817.

[15] H. Lozano , G. Catalán , J. Esteve , N. Domingo , G. Murillo , Nanotechnology 2020, 32, 025202. 10.1088/1361-6528/abb972 32942269

[16] A. Blanquer , O. Careta , L. Anido‐Varela , A. Aranda , E. Ibáñez , J. Esteve , C. Nogués , G. Murillo , Int. J. Mol. Sci. 2022, 23, 432. 10.3390/ijms23010432 PMC8745485 35008860

[17] G. S. Pitt , M. Matsui , C. Cao , Annu. Rev. Physiol. 2021, 83, 183. 33106102 10.1146/annurev-physiol-031620-091043 PMC8281591

[18] Q. Kang , E. J. Lang , M. Sahin , Front Neurosci. 2023, 17, 1282322. 38027520 10.3389/fnins.2023.1282322 PMC10667418

[19] B. Avlar , R. Rahman , S. Vendidandi , E. Cetinkaya , A. S. Asan , M. Sahin , E. J. Lang , Front Syst. Neurosci. 2023, 17, 1173738. 37274077 10.3389/fnsys.2023.1173738 PMC10232809

[20] A. Broncel , R. Bocian , P. Kłos‐Wojtczak , J. Konopacki , Brain Res. Bull. 2019, 152, 236. 31351158 10.1016/j.brainresbull.2019.07.025

[21] K. Maeda , R. Maruyama , T. Nagae , M. Inoue , T. Aonishi , H. Miyakawa , PLoS One 2015, 10, e0122263. 25811836 10.1371/journal.pone.0122263 PMC4374834

[22] D. Bironaite , J. Petroniene , R. Miksiunas , A. Zinovicius , I. Morkvenaite‐Vilkonciene , A. Ramanavicius , Electrochim. Acta 2023, 455, 142389.

[23] M. E. Buenning , M. Bielfeldt , B. Nebe , S. Staehlke , Appl. Sci. 2024, 14, 812.

[24] S. Staehlke , M. Bielfeldt , J. Zimmermann , M. Gruening , I. Barke , T. Freitag , S. Speller , U. Van Rienen , B. Nebe, Cells 2022, 11, 2650 10.3390/cells11172650 PMC9454840 36078058

[25] M. Bielfeldt , K. Budde‐Sagert , N. Weis , M. Buenning , S. Staehlke , J. Zimmermann , N. Arbeiter , S. Mobini , M. U. González , H. Rebl , A. Uhrmacher , U. van Rienen , B. Nebe , J. Biol. Eng. 2023, 17, 71. 37996914 10.1186/s13036-023-00393-1 PMC10668359

[26] M. Hu , H. Li , K. Zhu , L. Guo , M. Zhao , H. Zhan , P. N. Devreotes , Q. Qing , Sci Rep 2024, 14, 3167. 38326365 10.1038/s41598-024-53018-y PMC10850077

[27] L. Guo , H. Li , Y. Wang , Z. Li , J. Albeck , M. Zhao , Q. Qing , Nano Lett. 2019, 19, 7526. 31487192 10.1021/acs.nanolett.9b03411 PMC6786939

[28] E. Fernández‐Rosas , R. Gómez , E. Ibañez , L. Barrios , M. Duch , J. Esteve , J. A. Plaza , C. Nogués , Biomed. Microdev. 2010, 12, 371. 10.1007/s10544-009-9393-6 20069375

[29] T. Patiño , J. Soriano , L. Barrios , E. Ibáñez , C. Nogués , Sci Rep. 2015, 5, 11371. 26068810 10.1038/srep11371 PMC5155550

[30] R. Gómez‐Martínez , P. Vázquez , M. Duch , A. Muriano , D. Pinacho , N. Sanvicens , F. Sánchez‐Baeza , P. Boya , E. J. De La Rosa , J. Esteve , T. Suárez , J. A. Plaza , Small 2010, 6, 499. 20025079 10.1002/smll.200901041

[31] S. Novo , O. Penon , L. Barrios , C. Nogués , J. Santaló , S. Durán , R. Gómez‐Matínez , J. Samitier , J. A. Plaza , L. Pérez‐García , E. Ibáñez , Hum. Reprod. 2013, 28, 1519. 23532322 10.1093/humrep/det083

[32] F. Xiao , J. Li , K. Huang , X. Li , Y. Xiong , M. Wu , L. Wu , W. Kuang , S. Lv , L. Wu , X. Zhu , H. Guo , Am. J. Cancer Res. 2021, 11, 14. 33520357 PMC7840718

[33] E. Rainero , Curr. Opin. Cell Biol 2024, 88, 102359. 38626703 10.1016/j.ceb.2024.102359

[34] N. Torras , J. P. Agusil , P. Vázquez , M. Duch , A. M. Hernández‐Pinto , J. Samitier , E. J. De La Rosa , J. Esteve , T. Suárez , L. Pérez‐García , J. A. Plaza , Adv. Mater. 2016, 28, 1449. 26649987 10.1002/adma.201504164

