# Supplementary material for: Tailoring Piezoelectric Nanogenerators and Microdevices for Cellular Excitation: Impact of Size and Morphology
Source: Adv Sci (Weinh). 2025 Feb 14;12(24):2415028. doi: 10.1002/advs.202415028 (PMC12199572; doi:10.1002/advs.202415028)
Supplement: Supplementary file 1 — Supporting Information [file ADVS-12-2415028-s001.pdf]

ADVANCED SCIENCE
Open Access

Supporting Information

for *Adv. Sci.*, DOI 10.1002/advs.202415028

Tailoring Piezoelectric Nanogenerators and Microdevices for Cellular Excitation: Impact of Size and Morphology

Laura Lefaix, Marc Navarro, Carme Nogués, Andreu Blanquer and Gonzalo Murillo**

Supporting Information:

Tailoring piezoelectric nanogenerators and microdevices for cellular excitation: Impact of size and morphology

Laura Lefaix, Marc Navarro, Carme Nogués, Andreu Blanquer, Gonzalo Murillo**

The observation of increasing heterogeneity in samples with longer hydrothermal growth times and thicker AlN layers led to a further study on the distribution of ZnO NS thicknesses in the sample. For this, thickness values were evaluated in histogram plots shown in **Supplementary Figure S1**. In this figure it can be observed that shorter hydrothermal growth and thinner AlN layers promote more homogeneous samples (acute normal distribution curve, in red).

Homogeneity in the sample is necessary to achieve reproducibility in the cell response. Heterogeneous samples, such as those grown for 21 h and with a 500 nm AlN layer, may produce varying responses in the cells. The ones growth on thicker ZnO NSs may not be excited due to the inability to bend these ZnO NSs.

In **Supplementary Figure S2**, ZnO NSs after 3 h of hydrothermal growth using different drying methods were compared. It was first observed that ZnO NSs in the samples had collapsed at some areas, indicating the ethanol rinsing was insufficient. Thus, the use of HMDS, an effective reagent for fixation and dehydration of specimen for SEM, allowed us to obtain dry samples after 3 h of hydrothermal growth avoiding NS collapse. This enabled more accurate measurements of the ZnO NSs thickness under these conditions (Figure 2B), resulting in thinner NSs.

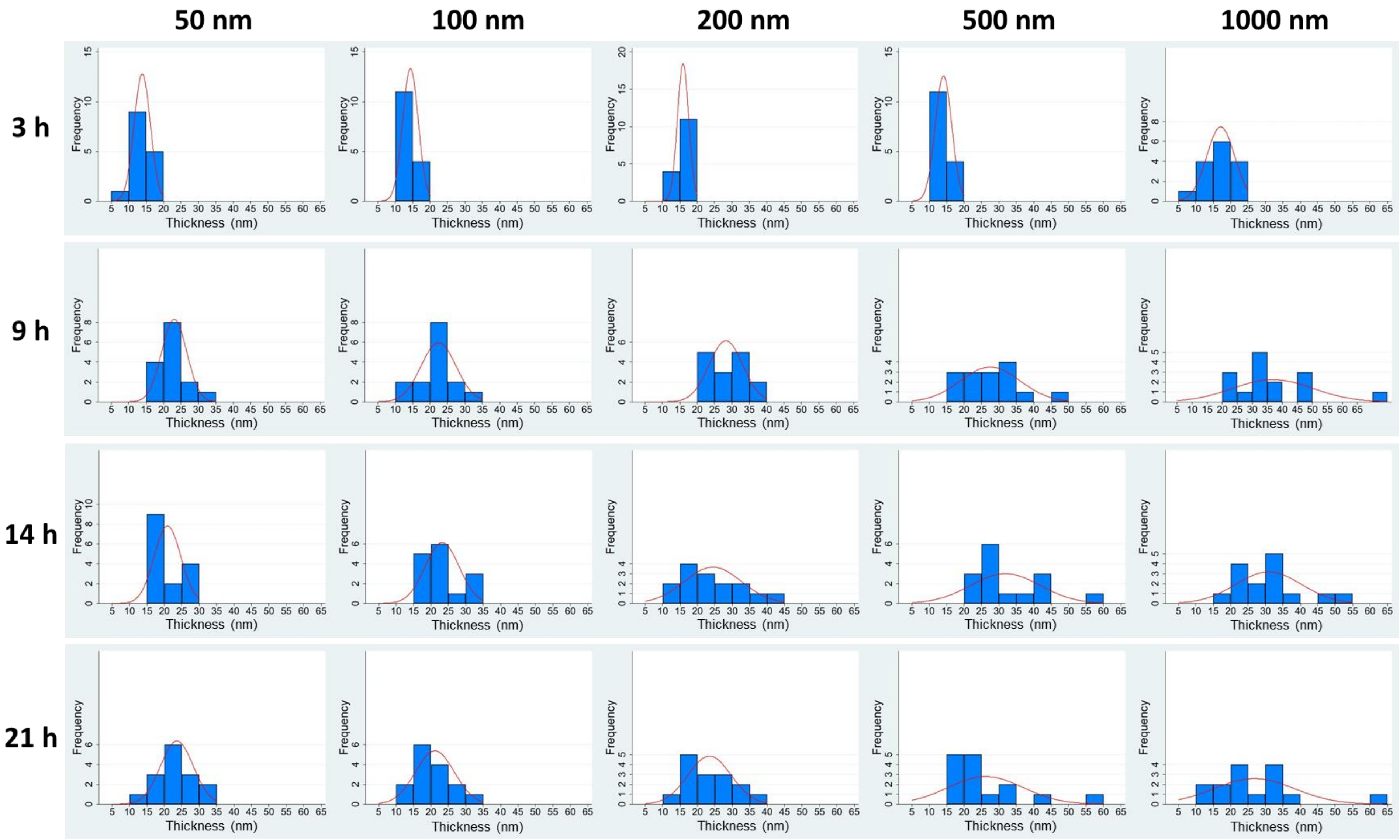
50 nm
100 nm
200 nm
500 nm
1000 nm
3 h
9 h
14 h
21 h
Frequency
Thickness (nm)

Supplementary Figure S1. Histograms depicting ZnO NS thickness distribution per sample. Every condition tested for hydrothermal growth times (3, 9, 14, 21 h) and AlN layer thickness (50, 100, 200, 500 and 1000 nm) was evaluated. The samples grown for 3 h were analyzed based on the results obtained after drying with HMDS. The normal distribution of the values for each sample is shown in red.

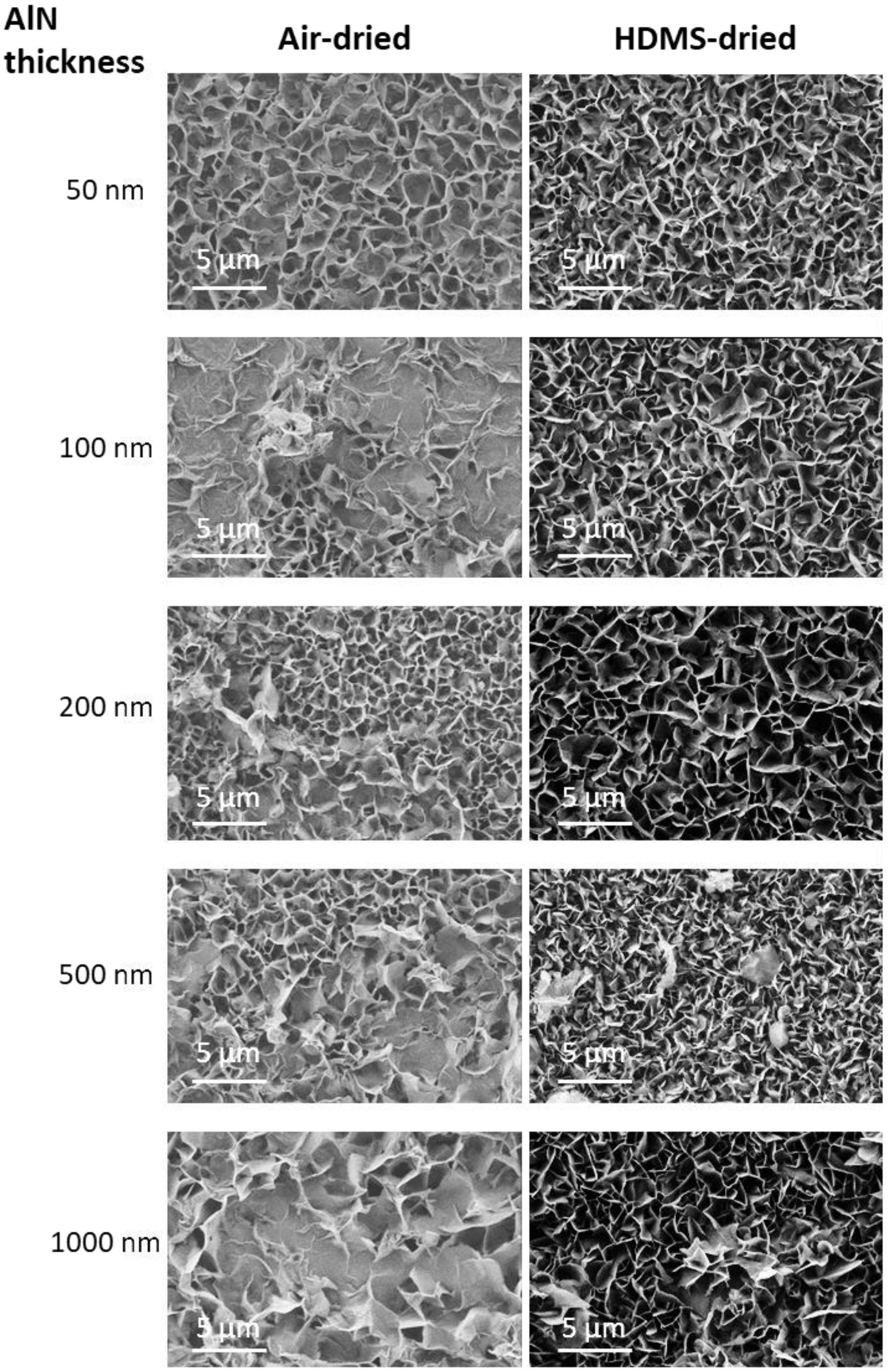

Supplementary Figure S2. SEM images of samples with different AlN thicknesses (50, 100, 200, 500, and 1000 nm) after 3 h of hydrothermal growth, comparing drying conditions. In the first column, showing ZnO NSs collapsed, the samples were rinsed with ethanol and left to dry. In the second column, where ZnO NSs did not collapse, the samples were dried using HMDS for 45 min.

The correct release of each type of microdevice was corroborated after observing the detachment of the microdevices from the silicon wafers. As depicted in **Supplementary Figure S3**, after the peel-off step, only the SiO_2 micropillar can be observed on the wafers.

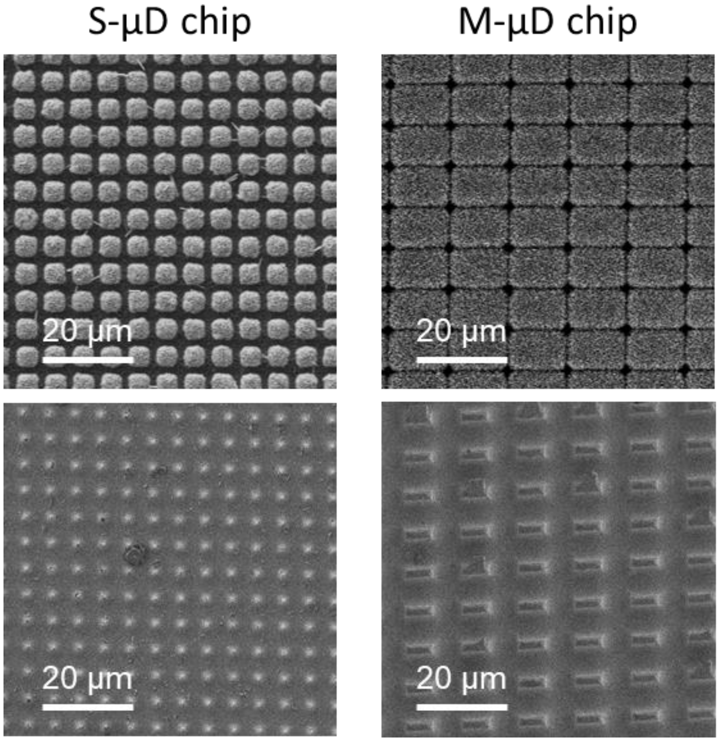

Supplementary Figure S3. SEM images of the silicon substrate wafers with microdevices before and after the peel-off step. Before the peel-off step (first row) the microdevices are anchored to the substrate through an etched pillar of SiO_2 under the microdevice. After the peeling-off (second row), the micropillars where the microdevices were standing can be observed on the silicon substrate, indicating that the microdevices remained embedded in the polymer for subsequent resuspension after the polymer is dissolved.
